# Whole-Genome Sequencing of Gram-Negative Bacteria Isolated From Bovine Mastitis and Raw Milk: The First Emergence of Colistin *mcr*-*10* and Fosfomycin *fosA5* Resistance Genes in *Klebsiella pneumoniae* in Middle East

**DOI:** 10.3389/fmicb.2021.770813

**Published:** 2021-12-08

**Authors:** Yasmine H. Tartor, Norhan K. Abd El-Aziz, Rasha M. A. Gharieb, Hend M. El Damaty, Shymaa Enany, Enas A. Soliman, Samah S. Abdellatif, Amira S. A. Attia, Mosa M. Bahnass, Yousry A. El-Shazly, Mohammed Elbediwi, Hazem Ramadan

**Affiliations:** ^1^Microbiology Department, Faculty of Veterinary Medicine, Zagazig University, Zagazig, Egypt; ^2^Zoonoses Department, Faculty of Veterinary Medicine, Zagazig University, Zagazig, Egypt; ^3^Animal Medicine Department (Infectious Diseases), Faculty of Veterinary Medicine, Zagazig University, Zagazig, Egypt; ^4^Microbiology and Immunology Department, Faculty of Pharmacy, Suez Canal University, Ismailia, Egypt; ^5^Bacteriology, Immunology and Mycology Department, Faculty of Veterinary Medicine, Benha University, Benha, Egypt; ^6^Food Control Department, Faculty of Veterinary Medicine, Zagazig University, Zagazig, Egypt; ^7^Veterinary Public Health Department, Faculty of Veterinary Medicine, Zagazig University, Zagazig, Egypt; ^8^Veterinary Hospital, Faculty of Veterinary Medicine, Zagazig University, Zagazig, Egypt; ^9^Animal Health Research Institute, Agriculture Research Center, Cairo, Egypt; ^10^Institute of Veterinary Sciences, Department of Veterinary Medicine, College of Animal Sciences, Zhejiang University, Hangzhou, China; ^11^Hygiene and Zoonoses Department, Faculty of Veterinary Medicine, Mansoura University, Mansoura, Egypt; ^12^Bacterial Epidemiology and Antimicrobial Resistance Research Unit, US National Poultry Research Center, US Department of Agriculture, Agricultural Research Service (USDA-ARS), Athens, GA, United States

**Keywords:** Gram-negative bacteria, mastitis, *Klebsiella pneumoniae*, *mcr-10*, *fosA5*, whole-genome sequencing

## Abstract

Antimicrobial resistance is a major concern in the dairy industry. This study investigated the prevalence, antimicrobial resistance phenotypes, and genome sequencing of Gram-negative bacteria isolated from clinical (*n* = 350) and subclinical (*n* = 95) bovine mastitis, and raw unpasteurized milk (*n* = 125). *Klebsiella pneumoniae*, *Aeromonas hydrophila*, *Enterobacter cloacae* (100% each), *Escherichia coli* (87.78%), and *Proteus mirabilis* (69.7%) were the most prevalent multidrug-resistant (MDR) species. Extensive drug-resistance (XDR) phenotype was found in *P. mirabilis* (30.30%) and *E. coli* (3.33%) isolates. Ten isolates (four *E. coli*, three *Klebsiella* species and three *P. mirabilis*) that displayed the highest multiple antibiotic resistance (MAR) indices (0.54–0.83), were exposed to whole-genome sequencing (WGS). Two multilocus sequence types (MLST): ST2165 and ST7624 were identified among the sequenced *E. coli* isolates. Three *E. coli* isolates (two from clinical mastitis and one from raw milk) belonging to ST2165 showed similar profile of plasmid replicon types: IncFIA, IncFIB, IncFII, and IncQ1 with an exception to an isolate that contained IncR, whereas *E. coli* ST7624 showed a different plasmid profile including IncHI2, IncHI2A, IncI1α, and IncFII replicon types. ResFinder findings revealed the presence of plasmid-mediated colistin *mcr*-*10* and fosfomycin *fosA*5 resistance genes in a *K. pneumoniae* (K1) isolate from bovine milk. Sequence analysis of the reconstructed *mcr-10* plasmid from WGS of K1 isolate, showed that *mcr-10* gene was bracketed by *xer*C and insertion sequence IS26 on an IncFIB plasmid. Phylogenetic analysis revealed that K1 isolate existed in a clade including *mcr-10*-harboring isolates from human and environment with different STs and countries [United Kingdom (ST788), Australia (ST323), Malawi (ST2144), Myanmar (ST705), and Laos (ST2355)]. This study reports the first emergence of *K. pneumoniae* co-harboring *mcr*-*10* and *fosA5* genes from bovine milk in the Middle East, which constitutes a public health threat and heralds the penetration of the last-resort antibiotics. Hence, prudent use of antibiotics in both humans and animals and antimicrobial surveillance plans are urgently required.

## Introduction

Mastitis is one of the most prevalent diseases in dairy cows, producing well-recognized detrimental effects on animal health and profitability of dairy farms (Ruegg, 2017; Abd El-Aziz et al., 2021a). Treatment of mastitis is often applied without prior knowledge of cause-related agents and mostly with broad-spectrum antimicrobials, which are known to enhance resistance evolution to a greater extent. This increases the selective pressure on potentially present pathogens and is considered a possible human health hazard (Neyra et al., 2014; Abd El-Aziz et al., 2021b).

Raw or processed milk is a good environment that promotes the existence of many pathogens, which could be either directly obtained via dairy cows, or indirectly from the environment of the farm (Oliver et al., 2005). *Escherichia coli* (*E. coli*) O157:H7 outbreaks associated with the consumption of raw milk have been described (Disassa et al., 2017; Ababu et al., 2020). The indiscriminate use of antimicrobials has led to the development of multidrug-resistant (MDR) Gram-negative bacteria in raw milk, in particular *E. coli* O157:H7, *Klebsiella pneumoniae* (*K. pneumoniae), Aeromonas hydrophilia* (*A. hydrophilia*), and *Proteus mirabilis* (*P. mirabilis*) (Zelalem and Bernard, 2006; Oliver et al., 2009; Garedew et al., 2012).

Resistance to most antimicrobial classes and the lack of new antimicrobial agents against Gram-negative bacteria potentiates the re-use of old antibiotics, particularly polymyxins such as colistin (Biswas et al., 2012). Colistin is currently known as the last-resort antimicrobial agent for the treatment of infections caused by MDR Gram-negative bacteria (Poirel et al., 2017). It binds to the negatively charged lipopolysaccharide (LPS) of the outer membrane of Gram-negative bacteria, leading to disruption of the membrane, induces the cytoplasmic material to fade away, and eventually cell death (Yahav et al., 2012).

Colistin has been greatly utilized in the agricultural system and veterinary medicine for decades, as a growth promoter and for the treatment of enterobacterial infections (Catry et al., 2015). Extensive colistin usages in livestock have facilitated the rapid spread of the *mcr* resistance genes (Shen et al., 2016). The *mcr-1* plasmid-mediated colistin resistance gene has been first reported in Enterobacterales isolated from animals, food, and humans in China (Liu et al., 2016). Numerous studies have documented plasmid-borne *mcr* variants (*mcr-1* to *mcr-9*) from this time onward (Xavier et al., 2016; Yin et al., 2017; Borowiak et al., 2019; Carroll et al., 2019; Gelbíčová et al., 2019; Wang et al., 2019). However, studies focused on *mcr*-resistant isolates from mastitis and raw unpasteurized milk have received little attention. Here, the genome sequences and genetic relatedness between MDR and extensive drug-resistant [XDR; resistance to at least one antibiotic in all tested antimicrobial groups except one or two as demonstrated in the worksheet of Magiorakos et al. (2012)]. Gram-negative bacteria isolated from bovine milk samples have been studied for the first time. This study aimed to: (1) investigate the occurrence and resistance phenotypes of the Gram-negative bacteria in mastitic cow’s milk as well as raw unpasteurized milk that consumed by the public; (2) identify the multilocus sequence types (MLST), resistance genes, and plasmid replicon types of MDR and XDR Gram-negative isolates; (3) determine the genetic environment of *mcr*-*10* gene and epidemiological relatedness between the study isolate and the publicly available genomes of *K. pneumoniae* isolates harboring *mcr* genes.

## Materials and Methods

### Milk Samples

This study was carried out during the period between October 2018 and August 2020. A total of 445 quarter milk samples were collected from lactating cows in smallholder farms with clinical (*n* = 350) and subclinical (*n* = 95) mastitis. Moreover, 125 raw unpasteurized milk samples were collected from retail milk outlets located in Zagazig City, Sharkia Governorate, Egypt. Clinical mastitis was diagnosed based on the cardinal signs of inflammation and the changes in the milk of the infected mammary quarters. California mastitis test was performed on apparently normal milk samples for diagnosis of subclinical mastitis. The milk samples were aseptically collected from mastitic dairy cows before the antibiotic therapy following the standard procedures (National Mastitis Council, 1999). Collected milk samples were labeled, kept in an icebox, and transferred immediately to the Microbiology laboratory for further bacteriological analysis.

### Isolation and Identification of Gram-Negative Bacteria

Ten milliliters of each milk sample were enriched in 90 mL of Buffered Peptone Water (BPW, Oxoid, United Kingdom) and incubated overnight at 37°C. A loopful from the enriched broth was cultivated on the agar media selective for each expected bacterium; coliforms (MacConkey Agar, CM0007; Oxoid, United Kingdom), *E. coli* (Eosin Methylene Blue Agar, CM0069; Oxoid, United Kingdom), *K. pneumoniae* (HiCrome Klebsiella Selective Agar, M1573; Himedia, India), and *Proteus* species [MacConkey Agar (without salt), CM0507; Oxoid, United Kingdom]. A suspected colony of each microorganism was purified on a slope agar, identified by Gram staining, and subjected to standard biochemical tests recommended for identification of each bacterial species according to Faddin (2000). Serotyping of all biochemically confirmed *E. coli* isolates was done by slide agglutination technique using diagnostic polyvalent and related monovalent *E. coli* O and H antisera (Denka Seiken Co., Japan). Polymerase chain reaction (PCR) was applied for confirmation of Gram-negative isolates using species-specific primer sets listed in Supplementary Table 1 (Cascón et al., 1996; Heijnen and Medema, 2006; Liu et al., 2008; Anbazhagan et al., 2010; Zhang et al., 2013). PCR amplifications were performed using a programmable 2720 thermal cycler (Applied Biosystem, Waltham, Massachusetts, United States) in a total reaction volume of 25 μL containing 12.5 μL of EmeraldAmp Max PCR Master Mix (Takara, Japan), 1 μL of each primer (20 pmoL) (Sigma-Aldrich, Co., St. Louis, United States), 5 μL of template DNA and 5.5 μL of nuclease-free water. The amplified products were visualized after 30 min of electrophoresis on a 2% agarose gel containing ethidium bromide.

### Antimicrobial Susceptibility Testing of Gram-Negative Bacteria

The antibiogram of all recovered Gram-negative bacteria was determined by the disc diffusion method (Bauer et al., 1966). The antimicrobial discs (Oxoid, Cambridge, United Kingdom) included ampicillin (AM; 10 μg), amoxicillin-clavulanic acid (AMC; 30 μg), piperacillin-tazobactam (TPZ; 40 μg), cefazolin (CFZ; 30 μg), cephalothin (CEF; 30 μg), cefoxitin (FOX; 30 μg), ceftriaxone (CRO; 30 μg), ceftazidime (CAZ; 30 μg), cefotaxime (CTX; 30 μg), cefepime (FEP; 30 μg), imipenem (IPM; 10 μg), gentamicin (CN; 10 μg), tobramycin (TOB; 10 μg), amikacin (AK; 30 μg), nalidixic acid (NA; 30 μg), ciprofloxacin (CIP; 5 μg), levofloxacin (LVX; 5 μg), sulfamethoxazole-trimethoprim (SXT; 25 μg), tigecycline (TGC; 15 μg), aztreonam (ATM; 30 μg), chloramphenicol (CHL; 30 μg), fosfomycin (FOF; 50 μg), colistin (CST; 25 μg), and tetracycline (TET; 30 μg). Selected antimicrobial agents were synchronized with the veterinary guidelines (Plumb, 2015). The justification for the antimicrobials chosen is to monitor antimicrobial resistance and for public health concerns about certain agents such as cephalosporins, carbapenems, aminoglycosides, fluoroquinolones, tigecycline, and fosfomycin. *E. coli* ATCC 25922, *K. pneumoniae* ATCC 700603, *P. mirabilis* ATCC 29906, and *A. hydrophila* ATCC 7966 were included as quality control strains. The minimum inhibitory concentration (MIC) for colistin against Gram-negative isolates was performed using broth microdilution method as documented previously (Rankin, 2005). The results for antimicrobial susceptibility testing were interpreted following the guidelines of the Clinical and Laboratory Standards Institute (CLSI, 2017) and the European Committee on Antimicrobial Susceptibility Testing guidelines (Eucast: The European Committee on Antimicrobial Susceptibility Testing, 2018). Colistin resistance breakpoint was considered at MIC > 2 mg/L. The fully colistin-susceptible *E. coli* ATCC 25922 and the *mcr-1*-positive *E. coli* NCTC 13846 with a colistin MIC of 4 mg/L were used as quality controls. The multiple antibiotic resistance (MAR) indices were estimated as documented by Tambekar et al. (2006), whereas the MDR and XDR isolates were determined according to Magiorakos et al. (2012).

### Whole-Genome Sequencing and Bioinformatics Analyses

Whole-genome sequencing (WGS) was performed for 10 representative MDR and XDR isolates that exhibited unique antibiogram and the highest MAR indices. DNA libraries were constructed using Nextra DNA Flex kit (Illumina Inc., San Diego, CA, United States), and libraries were then sequenced at a 250 bp paired-end-read format using Illumina NovaSeq 6000 kit (Illumina Inc., San Diego, CA, United States), according to the manufacturer’s instructions.

The obtained raw fastq reads for each isolate were deposited into sequence read archive (SRA) database under the bioproject PRJNA713498. Quality check of the fastq reads was performed using fastQC,^1^ and low-quality reads were exposed to trimmomatic^2^ (Bolger et al., 2014). The reads were then *de novo* assembled using SPAdes^3^ (Bankevich et al., 2012) and the assembled fasta files were uploaded into the Center for Genomic Epidemiology (CGE)^4^ to identify the MLST, resistance genes, and plasmid replicon types via MLST 2.0, ResFinder 4.1 and PlasmidFinder 2.1 tools, respectively. For genotyping and source tracking, MLST for *mcr-10*-producing *K. pneumoniae* isolate (K1) was determined using bacterial whole-genome sequence typing database (BacWGSTdb)^5^ (Ruan and Feng, 2016). The genetic environment of *mcr*-*10* gene was determined using a nucleotide Basic Local Alignment Search Tool (BLASTn) search^6^ in combination with ISFinder.^7^ The contig carrying the *mcr*-*10* was annotated using prokka, and the gbk file was imported into Easyfig software^8^ for genetic comparison (Sullivan et al., 2011). The *mcr-10*-carrying plasmid of the study isolate (K1) was reconstructed from WGS using Plasmid SPAdes (Antipov et al., 2016) and PLACNETw (Vielva et al., 2017). BLASTn search of the obtained plasmid sequences was performed to determine the best plasmid hits. Reconstructed plasmid sequences of K1 isolate were aligned to the retrieved National Center for Biotechnology Information (NCBI) plasmid sequences using BLAST Ring Image Generator (BRIG) tool^9^ for genetic comparison (Alikhan et al., 2011).

Phylogenetic analysis based on single nucleotide polymorphisms (SNPs) was performed for the *K. pneumoniae* isolate (K1) carrying *mcr-10* gene. Our K1 isolate was compared to the publicly available genomes of *K. pneumoniae* in the NCBI database, updated March 21^st^, 2021, that carried any variant of *mcr* gene (*n* = 241) or those carrying *mcr-10* variant (*n* = 20). Isolates were mapped to the reference *K. pneumoniae* complete genome ATCC43816 (CP064352.1) for all the enrolled *mcr*-carrying isolates and 30684/NJST258_2 (CP006918.1) for *mcr-10*-producing *K. pneumoniae* isolates. Metadata for the selected *K. pneumoniae* sequences from NCBI are listed in Supplementary Tables 2A,B. SNPs variant calling were determined using Snippy v4.4.4,^10^ and the output files were combined into a core SNPs alignment using Snippy core. Maximum likelihood phylogenetic trees were then generated from SNPs alignment using RAxML, and the trees were visualized with iTOL (Letunic and Bork, 2016).

## Results

### Prevalence of Gram-Negative Bacteria Isolated From Cattle Mastitis and Raw Milk

As depicted in Table 1, a total of 184 isolates were recovered from cases of clinical (120/350; 34.29%) and subclinical (19/95; 20%) cattle mastitis, and raw milk samples (45/125; 36%). PCR of the amplified products of bacterial species could identify the isolates as *E. coli* (48.91%), *K. pneumoniae* (13.59%), *P. mirabilis* (17.93%), *A. hydrophila* (9.24%), *E. cloacae* (8.15%), and *C. freundii* (2.17%). According to the serotyping results, a variety of *E. coli* serotypes (*n* = 10) was identified. The predominant serotypes were O111:H4 from clinical (8%) and subclinical (2.11%) mastitis and O157:H7 from raw milk (6.4%).

**TABLE 1:** Prevalence of Gram-negative bacteria isolated from clinical, subclinical bovine mastitis, and raw milk in Egypt.

Bacterial species (No.)	No. of isolated bacteria (%)			No. of MDR isolates (%)	No. of XDR isolates (%)
	Clinical mastitis (*n* = 350)	Subclinical mastitis (*n* = 95)	Raw milk (*n* = 125)		
*E. coli* (90):	73 (20.86)*	6 (6.32)	11 (8.8)	79 (87.78)	3 (3.33)
O111:H4	28 (8)	2 (2.11)	0		
O114:H21	13 (3.71)	0	0		
O114:H4	0	1 (1.05)	0		
O146:H−	12 (3.43)	0	0		
O26:H11	11 (3.14)	0	2 (1.6)		
O−:H7	3 (0.86)	0	1 (0.8)		
O157:H7	3 (0.86)	1 (1.05)	8 (6.4)		
O44:H18	2 (0.57)	1 (1.05)	0		
O119:H6	0	1 (1.05)	0		
O−: H34	1 (0.29)	0	0		
*K. pneumoniae* (25)	12 (3.43)	4 (4.21)	9 (7.2)	25 (100)	0
*Enterobacter cloacae* (15)	5 (1.43)	2 (2.11)	8 (6.4)	15 (100)	0
*Citrobacter freundii* (4)	4 (1.14)	0	0	1 (25)	0
*Proteus mirabilis* (33)	26 (7.43)	7 (7.37)	0	23 (69.7)	10 (30.30)
*Aeromonas hydrophila* (17)	0	0	17 (13.6)	17 (100)	0
Total (184)	120 (34.29)	19 (20)	45 (36)	160 (86.96)	13 (7.07)

*Percentage was calculated from the examined sample source; MDR, multidrug-resistant; XDR, extensive drug-resistant.

### Frequency of Multidrug-Resistant and Extensive Drug-Resistant Gram-Negative Bacteria From Milk Samples

The MDR and XDR phenotypes were determined in 86.96 and 7.07% of the isolates, respectively (Table 1). *K. pneumoniae*, *A. hydrophila*, *E. cloacae* (100%, each), *E. coli* (87.78%), and *P. mirabilis* (69.7%) were the most prevalent MDR species. XDR phenotype was found in *P. mirabilis* (30.30%) and *E. coli* (3.33%).

The most observed resistance phenotypes for *E. coli* isolates were against cephalothin, cefazolin, ceftazidime, ampicillin, amoxicillin-clavulanic acid (100% each), cefoxitin (98. 89%), cefotaxime (94.44%), piperacillin-tazobactam (75.56%), fosfomycin (63.33%), and colistin (26.67%). *K. pneumoniae* isolates were resistant to cephalothin, cefazolin, cefoxitin, ampicillin, amoxicillin-clavulanic acid, fosfomycin (100% each), ceftazidime, piperacillin-tazobactam (96% each), tigecycline (88%), cefotaxime and ceftriaxone (76% each), sulfamethoxazole-trimethoprim and colistin (56% each).

All *A. hydrophila* isolates were resistant to cephalothin, cefoxitin, ceftazidime, cefotaxime, ceftriaxone, and tetracycline. Meanwhile, 82.35, 76.47, and 70.59% of the isolates were resistant to colistin, nalidixic acid, and sulfamethoxazole-trimethoprim, respectively.

*Proteus mirabilis* isolates exhibited a resistance rate of 100% to cephalothin, cefazolin, cefoxitin, sulfamethoxazole-trimethoprim, amoxicillin-clavulanic acid, and fosfomycin followed by ampicillin, tetracycline, and chloramphenicol (90.91% each), nalidixic acid (78.79%), ciprofloxacin, and levofloxacin (60.61% each), whereas a lower resistance rate was reported for colistin (4/33; 12.12%). All *E. cloacae* isolates were resistant to cephalothin, cefazolin, cefoxitin, ceftazidime, cefotaxime, cefepime, sulfamethoxazole-trimethoprim, ampicillin, amoxicillin-clavulanic acid, and tetracycline. While, *C. freundii* isolates were resistant to cephalothin, cefazolin, cefoxitin, ceftazidime, cefotaxime, ampicillin, and amoxicillin-clavulanic acid (100%). The latter two bacterial species showed no colistin resistance.

### Whole-Genome Sequencing and Characteristics of Multidrug-Resistant and Extensive Drug-Resistant Gram-Negative Bacteria

We sequenced the genome of 10 representative Gram-negative bacterial isolates with the highest MAR indices (0.54–0.83) and determined the resistance genes, plasmid replicon types, and MLST types (Table 2). *In silico* analysis of *E. coli* sequences using MLST revealed that four isolates were assigned to two sequence types: ST2165 (*n* = 3) and ST7624 (*n* = 1). The three isolates belonging to ST2165 showed a markedly similar profile of plasmid replicon types including IncFIA, IncFIB, IncFII, and IncQ1 with an exception to isolate E2 that also contained IncR replicon type. The other *E. coli* isolate (E4) that was assigned to ST7624 showed the existence of IncHI2, IncHI2A, and IncI1α replicon types beside IncFII. Moreover, isolate E4 harbored the *bla*_*CTX*–*M*–14b_ gene that has not been identified in any of the other three *E. coli* ST2165 isolates. MLST analysis of the three-klebsiella isolates (two *K. pneumoniae* and one *K. aerogenes* as finally identified by WGS) assigned *K. aerogenes* (K2) and *K. pneumoniae* (K3) into ST11 and ST48, respectively, and *K. pneumoniae* (K1) into a novel ST, closely matches ST1224. Both *K. pneumoniae* isolates showed multiple replicon types, whereas *K. aerogenes* only carried IncR replicon type. The *K. pneumoniae* isolate (K1) that carried IncFIA(HI1), IncFIB(K), IncFII(K), IncHI1B(pNDM-MAR), IncQ1, Col(pHAD28), Col440I, and IncR replicon types, was found harboring multiple resistance genes to ß-lactamases, aminoglycosides, quinolones, tetracyclines, macrolides, folate pathway inhibitors as well as the last therapeutic choices such as fosfomycin (*fosA5*) and colistin (*mcr*-*10*). PlasmidFinder results displayed the existence of both IncQ1 and pSL483 replicon types in the three examined *P. mirabilis* isolates.

**TABLE 2:** Molecular characteristics of MDR and XDR Gram-negative bacteria recovered from milk samples.

Isolate ID/Species	Source	Antimicrobial resistance pattern	MAR index	Plasmid replicon types (Inc)	Sequence type (ST)	Resistance genes	Accession No.
**E1/*E. coli* (O−:H7)**	Clinical mastitis	AM, AMC, CEF, CRO, CAZ, CTX, FEP, CFZ, TPZ, TOB, CN, NA, CIP, LVX, SXT, ATM, CHL, TE, FOF	0.83	IncFIA, IncFIB(AP001918), IncFII, IncFII(p96A), IncFII(pCTU2), IncQ1, pESA2	ST2165	*fosA*, *ARR-2*, *sul1*, *sul2*, *ant(6)-Ia,aph(3″)-Ib*, *aph(6)-Id*, *aph(3′)-III*, *aph(4)-Ia*, *aac(3)-IIa*, *aadA7*, *ant(2″)-Ia*, *aadA2*, *aac(3)-IV*, *aadA1*, *aph(3′)-Ia, str*, *lnu*(F), *mdf*(A), *lsa*(A), *erm*(B), *erm*(42), *bla*_*TEM*–1B_, *bla*_*OXA*–10_, *bla*_*CMY*–4_, *bla*_*CMH*–3_, *qnrA1*, *cat*, *floR*, *dfrA14*, *dfrA1*, *dfrG*, *dfrA12*, *tet*(M), *tet*(C), *tet*(L), *tet*(A), *tet*(J)	SRR13933227
E2/*E. coli* (O−:H7)	Clinical mastitis	AM, AMC, CFZ, FOX, TPZ, CEF, CAZ, CTX, FOF, TET, CHL, TGC, SXT, TOB, CN	0.67	IncFIA, IncFIB(AP001918), IncFII, IncFII(p96A), IncQ1, IncR, pSL483	ST2165	*bla*_*CMY*4_, *tet*(*L*), *tet*(M), *tet*(J), *tet*(A), *fosA7*, *mdf*(A), *erm*(B), *erm*(42), *cat, floR*, *aac(3)-Iva*, *aadA2*, *aph(3″)-Ib, aac(6′)-Iaa*, *aph(4)-Ia*, *aph(3′)-Ia*, *aph(6)-Id*, *aadA1*, *ant(2″)-Ia, dfrA1*, *sul2*, *sul1*	SRR13933226
K2/*K. aerogenes*	Subclinical mastitis	AM, AMC, CFZ, CEF, FOX, CAZ, CTX, CRO, TPZ, TOB, TGC, TET, FOF, SXT, NA	0.63	IncR	ST11	*qnrS1*, *aph(3″)-Ib, tet*(A), *fosA*, *sul2*	SRR13933224
E4/*E. coli* (O−: H34)	Clinical mastitis	AM, AMC, CFZ, FOX, TPZ, CEF, CAZ, CTX, TGC, FOF, NA, CIP, SXT, TOB, TET, CHL	0.67	IncFII(p96A), IncHI2, IncHI2A, IncI1α	ST7624	*aadA1*, *aph(3′)-Ia, aac(6′)-Iaa*, *aadA2b*, *cmlA1*, *floR*, *erm*(42), *mdf*(A), *lnu*(F), *bla*_*CMY*–4_, *bla*_*TEM*–1B_, *bla*_*CTX*–*M*–14b_, *tet*(A), *sul3*, *gyrA* p.S83L	SRR13933223
K1/*K. pneumoniae*	Raw milk	AM, AMC, CFZ, CEF, FOX, CAZ, CTX, CRO, FEP, TPZ, FOF, NA, CIP, SXT, CST, TET, TOB, FOF, CHL	0.79	Col(pHAD28), Col440I, IncFIA(HI1), IncFIB(K), IncFII(K), IncHI1B(pNDM-MAR), IncQ1, IncR	NT*	*mcr-10*, *fosA5*, *bla*_*CMH*–3_, *bla*_*ACT*–16_, *bla*_*TEM*–1B_, *bla*_*LEN*26_, *bla*_*CMY*–4_, *bla*_*OXY*–4–1_, *bla*_*DHA*–1_, *bla*_*OKP*–*B*–2_, *bla*_*MIR*–3_, *qnrB4*, *qnrB1*, *qnrB19*, *dfrA1*, *dfrA14*, *sul1*, *sul2*, *tet(A)*, *aph(3″)-Ib*, *aph(6)-Id*, *aadA1*, *aph(3″)-Ib*, *catA1*	SRR13933222
E3/*E. coli* (O−:H7)	Raw milk	AM, AMC, CFZ, CEF, FOX, CAZ, CTX, NA, CIP, LVX, TET, SXT, CHL, AK	0.58	IncFIA, IncFIB(AP001918), IncFII, IncQ1, pSL483	ST2165	*mdf*(A), *erm*(42), *lnu*(F), *dfrA1*, *dfrA12*, *floR*, *cat*, *tet*(J), *tet*(A), *sul2*, *sul1*, *aph(6)-Id*, *aph(3″)-Ib, aadA1*, *aph(3′)-Ia*, *aadA2*, *aph(4)-Ia*, *aac(6′)-Iaa*, *aac(3)-IV*, *bla*_*CMY*–4_	SRR13933221
K3/*K. pneumoniae*	Clinical mastitis	AM, AMC, CFZ, CEF, FOX, CAZ, CTX, CRO, FEP, TPZ, SXT, TGC, FOF, NA, CIP, TET, CHL, CN	0.75	Col(IRGK), IncFIB(K), IncFII(K), IncR	ST48	*oqxA*, *oqxB*, *tet*(D), *catA2*, *mph*(A), *dfrA12*, *bla*_*TEM*–1D_, *bla*_*SHV*–172_, *fosA5*, *sul1*, *aph(3′)-Ia*, *aadA2*	SRR13933220
P2/*P. mirabilis*	Subclinical mastitis	AM, AMC, CEF, FOX, CAZ, CFZ, NA, SXT, CHL, FOF, TET, TOB, NA	0.54	IncQ1, pSL483	ND	*qnrA*1, *floR*, *cat*, *tet*(J), *tet*(A), *tet*(C), *ARR*-2, *bla*_*CMY*–2_, *bla*_*OXA*–10_, *bla*_*TEM*–1B_, *aph*(3′)-*Ia*, *aph*(3*″*)-*Ib*, *aadA*7, *aph*(6)-*Id*, *aac*(6′)-*Iaa*, *aac*(3)-*IIa*, *aadA*2, *ant*(2*″*)-*Ia*, *aac*(3)-*Id*, *aadA*1, *lnu*(F), *ere*(A), *sul*2, *sul1*, *dfrA32*, *dfrA14*, *dfrA1*	SRR13933219
P3/*P. mirabilis*	Subclinical mastitis	AM, AMC, CEF, FOX, CFZ, NA, CIP, LVX, SXT, CHL, FOF, TET, TOB, CN	0.58	Col(pHAD28), IncFII(Yp), IncQ1, pSL483	ND	*sul2*, *sul1*, *ARR* 2, *aadA*1, *floR*, *cat*, *tet*(A), *tet*(C), *aph*(3*″*)-*Ib*, *aph*(6)-*Id*, *aac*(3)-*IIa*, *aadA*7, *aph*(3′)-*Ia*, *aac*(3)-*IV*, *aadA*2*b*, *aph*(4)-*Ia*, *aadA1*, *aac(3)-Id*, *bla*_*TEM*–1B_, *bla*_*CMY*–4_, *bla*_*OXA*–10_, *lnu*(*F*), *erm*(42), *ere*(A), *dfrA14*, *dfrA12*, *dfrA1*, *dfrA32*, *qnrA*1	SRR13933218
**P4/*P. mirabilis***	Clinical mastitis	AM, AMC, CEF, FOX, CFZ, CRO, FEP, AK, CN, NA, CIP, LVX, SXT, TGC, CHL, FOF, TET	0.71	Col(pHAD28), IncQ1, pSL483	ND	*ARR-2*, *qnrA1*, *dfrA12*, *dfrA14*, *dfrA32*, *dfrA1*, *cat, floR*, *aadA2*, *ant(2″)-Ia*, *aadA7*, *aac(3)-Id*, *aac(3)-IV*, *aadA1*, *aph(3″)-Ib, aadA2*, *aph(3′)-Ia*, *aph(4)-Ia*, *aac(3)-IIa*, *aph(6)-Id*, *aac(6′)-Iaa*, *bla*_*TEM*–1B_, *bla*_*CMY*4_, *bla*_*OXA*–10_, *tet*(A), *tet*(C), *sul1*, *sul2*, *ere*(A), *erm*(42), *lnu(F)*	SRR13933225

All isolates are multidrug-resistant (MDR) except the bold ones are extensive drug-resistant (XDR) isolates. *NT, novel type closely matches ST1224. The seven alleles are: gapA (18), infB (118), mdh (26), pgi (63), phoE (142), rpoB (38), and tonB (169). Only one allele differs from ST1224. (https://bigsdb.pasteur.fr/klebsiella/). ND, not detected; AM, ampicillin; AMC, amoxicillin-clavulanic acid; TPZ, piperacillin-tazobactam; CFZ, cefazolin; CEF, Cephalothin; FOX, cefoxitin; CAZ, ceftazidime; CTX, cefotaxime; CRO, ceftriaxone; FEB, cefepime; CN, gentamicin; TOB, tobramycin; AK, amikacin; NA, nalidixic acid; CIP, ciprofloxacin; LVX, levofloxacin; TET, tetracycline; TIG, tigecycline; CHL, chloramphenicol; SXT, sulfamethoxazole-trimethoprim; ATM, aztreonam; CST, colistin; FOF, fosfomycin. CST resistance was determined based on the results of broth microdilution method (MIC resistance breakpoint > 2).

### Plasmid Characterization and Genetic Context of *mcr*-*10* Gene

**FIGURE 1:**
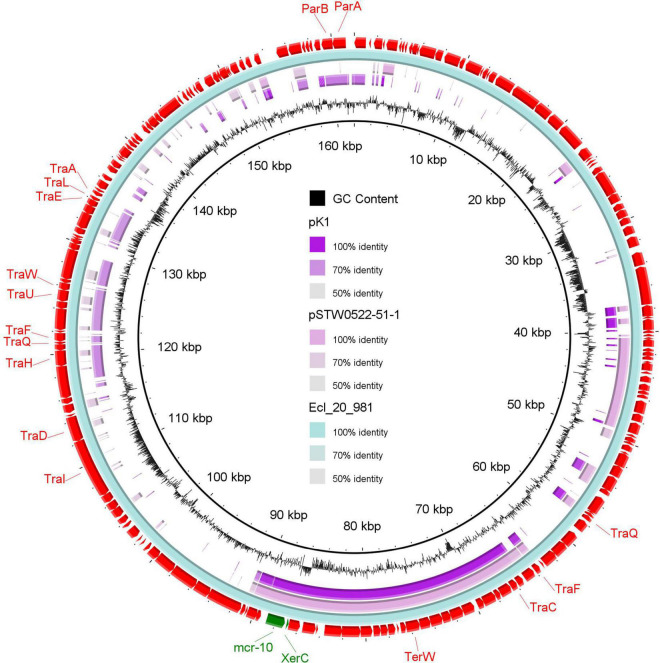
A circular map of the reconstructed IncFIB plasmid (pK1) from the draft genome sequence of *K. pneumoniae* (study isolate) compared to two NCBI retrieved plasmids, pEcl_20_981 of *Enterobacter roggenkampii* strain Ecl_20_981 (CP048651.1) and pSTW0522-51-1 of *Enterobacter kobei* strain STW0522-51 (AP022432.1). The out-layer circle (red color) represents plasmid pEcl_20_981, which used as the reference plasmid for sequence comparisons. The figure was generated using BLAST Ring Image Generator (BRIG) tool (http://sourceforge.net/projects/brig).

**FIGURE 2:**
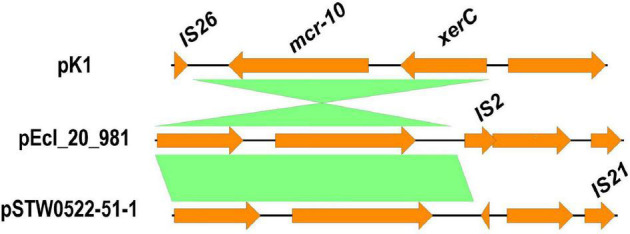
Schematic representation of the genetic environments of *mcr-10* identified from the draft genome sequence of *K. pneumoniae* isolate (K1). The figure was drawn using the EasyFig tool (http://mjsull.github.io/Easyfig/).

Plasmid reconstruction from the WGS of *K. pneumoniae* isolate (K1) using Plasmid SPAdes and PLACNETw revealed the existence of *mcr*-*10* gene on an IncFIB plasmid (pK1). BLASTn of the reconstructed pK1 plasmid showed sequence similarities with the backbone of other IncF plasmids like *Enterobacter kobei* STW0522-51 (AP022432.1) plasmid pSTW0522-51-1 and *Enterobacter roggenkampii* Ecl_20_981 (CP048651.1) plasmid pEcl_20_981 (Figure 1). Our pK1 plasmid and the two NCBI retrieved plasmids, pSTW0522-51-1 and pEcl_20_981, possessed transfer (*tra*) genes, which are responsible for the plasmid conjugation. Genetic mapping of *mcr*-*10* gene as depicted in Figure 2 revealed the existence of *mcr*-*10* flanked by *xer*C and IS26 in our K1 isolate, *xer*C and IS2 in isolate Ecl_20_981 (CP048651.1) and *xer*C and IS21 in isolate STW0522-51 (AP022432.1).

### Epidemiological Relatedness Between *mcr-10*-Harboring Isolate and the Publicly Available *Klebsiella pneumoniae* Isolates

**FIGURE 3:**
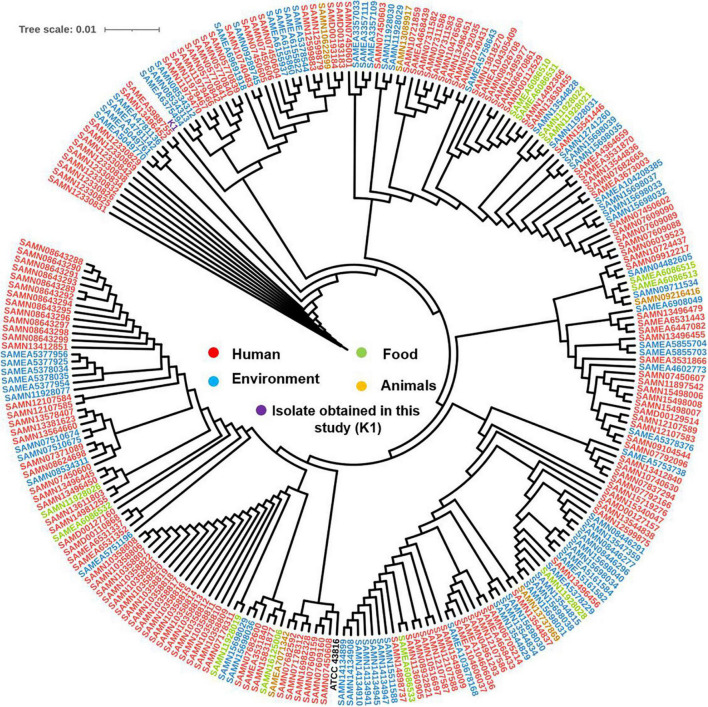
Global phylogenomic analysis of the present study *K. pneumoniae* isolate (K1) and all the publicly available genomes of *K. pneumoniae* (*n* = 241) in the NCBI database that carried any variant of *mcr* gene. Isolates were mapped to a reference *K. pneumoniae* complete genome ATCC 43816 (CP064352.1).

**FIGURE 4:**
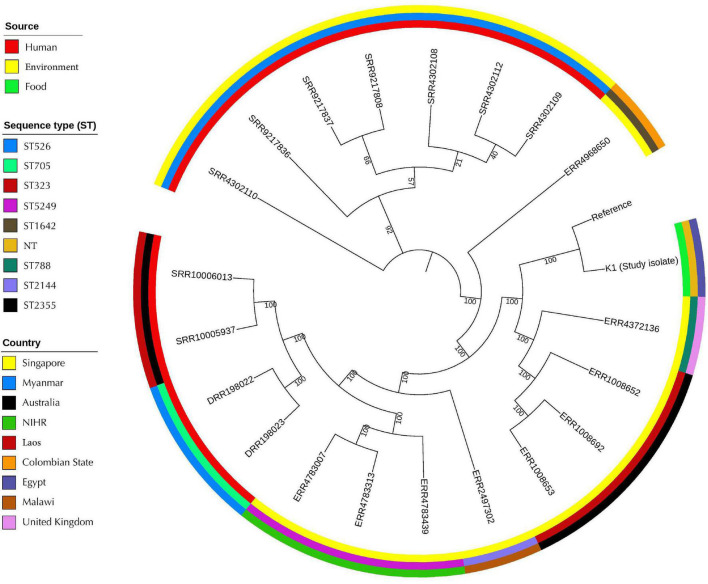
Global phylogenomic analysis of the present study *K. pneumoniae* isolate (K1) and the publicly available genomes of *mcr-*10-producing *K. pneumoniae* (*n* = 20) in the NCBI database. Isolates were mapped to a reference *K. pneumoniae* complete genome 30684/NJST258_2 (CP006918.1).

The SNPs-based phylogenetic analysis was performed to determine the epidemiological relatedness between the study isolate (K1) and the publicly available *K. pneumoniae* isolates (*n* = 241) harboring *mcr* genes in the NCBI database. The findings showed clustering of K1 isolate in a clade containing isolates (*n* = 25) from humans (clinical), environment, and animals from different countries, including China, Singapore, Thailand, United States, Laos, and Myanmar (Figure 3 and Supplementary Table 2A). Likewise, when comparing our isolate to *mcr-10*-producing *K. pneumoniae* isolates from NCBI, the findings revealed the existence of our K1 isolate in a clade encompassing isolates belonging to various STs and sourced from humans and environment from different countries [United Kingdom (ST788), Australia (ST323), Malawi (ST2144), Myanmar (ST705), and Laos (ST2355)] (Figure 4 and Supplementary Table 2B).

## Discussion

The development of resistance to numerous antimicrobial agents in pathogenic microbes has become a critical threat, as there are fewer or indeed no effective antimicrobial agents beneficial for treating these organisms. The recent global dissemination of MDR and XDR Gram-negative bacteria has raised the alarm of antimicrobial resistance as a serious and an urgent threat to public health. Consequently, studies have been conducted to explore the distribution of several resistance genes in different reservoirs (Tartor and El-Naenaeey, 2016; Elmowalid et al., 2018; Abd El-Aziz et al., 2021c).

Mastitis is considered one of the most frequent diseases in dairy cows in Egypt. Here, we studied MDR and XDR Gram-negative bacteria isolated from mastitic and raw unpasteurized milk. Many studies have confirmed the presence of Gram-negative bacteria in mastitic milk (Hogan and Larry Smith, 2003; Malinowski et al., 2006; Nam et al., 2009). Similarly, we identified a variety of Gram-negative bacteria from milk samples. The most frequent was *E. coli* (48.91%), followed by *P. mirabilis* (17.9%), while the least isolated one was *C. freundii* (2.1%). Comparably, Nam et al. (2009) declared that *E. coli* predominated the Gram-negative pathogens from milk samples, while *C. freundii* was the least frequent one. Our prevalence of Gram-negative bacteria in subclinical mastitis was lower than those reported earlier (Mahlangu et al., 2018). These variations in the prevalence could be attributed to the changes in the host, the management factors, and the environmental differences that impact the infection (Amin et al., 2013).

Interestingly, we reported 24.46% (45/184) of the isolated bacteria from raw unpasteurized milk, which directs our attention to the human being, especially when talking later about MDR and XDR isolates as well as colistin resistance. A recent study revealed the presence of Gram-negative bacteria in raw milk and other dairy products in Egypt and imputed this contamination to the evidence that most of Gram-negative bacteria constitute a part of the flora of the mouth and intestinal tract of humans and animals as well as their presence in soil, water, and sewage (Sobeih et al., 2020).

Multidrug-resistant isolates were known to be common in mastitis milk samples. Herein, *A. hydrophila*, *K. pneumoniae*, *E. cloacae* (100% each), *E. coli* (87.78%), and *P. mirabilis* (69.7%) were MDR. All MDR *A. hydrophila* isolates were isolated from raw milk. Previous reports declared that MDR *A. hydrophila* isolates were commonly isolated from water and fish samples (Igbinosa et al., 2013; Tartor et al., 2021a), which affirm the role of human beings and environment in transferring MDR *A. hydrophila* to the raw milk.

Similarly, although we identified all *E. cloacae* as MDR isolates, they were previously reported with a low frequency (7.1%) in milk samples from Egypt in 2011 (Ahmed and Shimamoto, 2011). However, they were recently detected in human samples (Huang et al., 2021), which in turn emphasizes again the concept of transferring between animal/animal products including milk from/to human and animal during these 10 years. Alternatively, MDR *K. pneumoniae* isolates were widely detected in milk samples (Yang et al., 2020) and this is concordant with what was reported in our study.

It is known that *E. coli* is the major pathogen causing environmental mastitis and the spreading of MDR *E. coli* is considered a key challenge in mastitis treatment, especially, when combined with an XDR phenotype (Basak et al., 2016). We identified 7.07% XDR *E. coli* and *P. mirabilis* isolates. XDR is considered a pop-up threat; the emergence of commonly XDR, carbapenem-resistant, and ESBL producing *E. coli* reviewing the abundant usage of the broad-spectrum antimicrobials. A former report on MDR and XDR *P. mirabilis* infections has revealed that aminoglycosides, penicillins, cephalosporins, carbapenems, and fluoroquinolones have become ineffective as first-line agents (Magiorakos et al., 2012). Serotyping of *E. coli* isolates showed 10 different serotypes. As expected from the previous reports, O111:H4 was the predominant type in clinical and subclinical mastitis, whereas O157:H7 was prevalent in raw milk (Denny et al., 2008; Sayed, 2014; Ababu et al., 2020).

Since the first colistin-resistant mutant that was reported in 1981 (Vaara et al., 1981), colistin resistance has been evolving in several microorganisms like *A. baumannii* (Potron et al., 2019), *K. pneumoniae* (Shankar et al., 2019; Tartor et al., 2021b), and *E. cloacae* (Huang et al., 2019). A recent study has reported *mcr*-harboring isolates from animals and humans (Luo et al., 2020) and another group has isolated *mcr*-positive *E. coli* and *K. pneumoniae* from cancerous patients in Egypt (Zafer et al., 2019). Concordant with previous studies, we reported *mcr*-10 harboring *K. pneumoniae*.

To go deeper in the genetic and molecular characterization of MDR and XDR Gram-negative isolates, we selected the isolates with the highest MAR indices for WGS analysis. *E. coli* were found to have two-allele sequence types; ST2165 and ST7624. Although ST2165 was previously detected in colistin-resistant *E. coli* carrying *mcr*-*1* from residents of long-term-care facilities (Giufrè et al., 2016), it was isolated elsewhere from chickens and store-bought produce (Reid et al., 2020; Foster-Nyarko et al., 2021). To our knowledge, this is the first isolation of this sequence type from milk. Similarly, *E. coli* ST7624 was not identified before from milk samples, although it was reported in wildlife (Arribas, 2019). In the view of plasmid replicon types, we found that the three *E. coli* isolates belonging to ST2165 have plasmid replicon types; IncFIA, IncFIB, IncFII, and IncQ1. Likewise, Foster-Nyarko et al. (2021) reported that the MDR isolates often co-carried large IncF plasmids like IncFIA, IncFIB, or IncFIC.

Herein, we found that *E. coli* isolates belonging to ST2165 are carrying several antibiotic resistance genes (Table 2), which confirms that IncF plasmids can play a potential role in disseminating antimicrobial resistance (Adenipekun et al., 2019). Comparably, the *E. coli* isolate belonging to ST7624 possessed multiple resistance genes to B-lactams (*bla*_*CMY*–4_, *bla*_*TEM*–1B_ and *bla*_*CTX*–*M*–14b_), aminoglycosides [*aadA*1, *aph(3′)*-*Ia*, *aac(6′)*-*Iaa* and *aadA2b*], tetracyclines [*tet*(A)], sulfonamides (*sul3*), macrolides (*erm*), lincosamide (*lnuF*), and phenicols (*floR*, *cmlA1*), that might be attributed to the presence of IncFII, IncHI2, IncHI2A, and IncI1α replicon types. In a previous study, 27 acquired antimicrobial resistance genes were located on the IncHI2-IncHI2A-type plasmid, including *bla_*CTX*–*M*–65_, fosA3, mphA, qepA*, and *rmtB* (Zhang et al., 2021).

On the other hand, WGS analysis of *Klebsiella* species showed that *K. pneumoniae* had a novel ST, that was closely matched to ST1224, and ST48 alleles. In earlier studies, *K. pneumoniae* ST1224 was isolated from neonates (Chen et al., 2019), while *K. pneumoniae* ST48 was reported in chicken meat in Western Algeria, one of the Middle East region (Chaalal et al., 2021) indicating that these STs are not host specific and could be easily transmitted to humans from food animals and their products.

On contrary, although we identified ST11 in *K. aerogenes*, a previous study found ST11 in *K. pneumoniae* (Hao et al., 2019).

Interestingly, this study is the first isolation of *mcr*-*10*-carrying *K. pneumoniae* isolate from a raw milk sample. The *mcr*-*10* gene flanked by *xer*C and IS26 in K1 isolate, *xer*C and IS2 in isolate Ecl_20_981 (CP048651.1) and *xer*C and IS21 in isolate STW0522-51 (AP022432.1). This possibly indicates the role of *xer*C gene, that encodes a tyrosine recombinase, in the mobilization of *mcr*-*10* gene (Wang et al., 2020; Xu et al., 2021). Although the *mcr*-*10* gene was detected recently in clinical strains of *Enterobacter roggenkampii* from different sources such as chickens (Lei et al., 2020), a patient (Wang et al., 2020), and sewage water (Xu et al., 2021), it is foremost found here in the raw milk sample that sequentially implicating the human in the transmission. Numerous isolates carrying *mcr-1* gene obtained from hard cheese made from raw milk were reported in Egypt (Hammad et al., 2019; Ombarak et al., 2021), which in turn highlights the importance of raw milk quality control check in Egypt focusing on farm workers as well as the animal itself.

Furthermore, our *mcr-10*-producing *K. pneumoniae* isolate was also carrying *fosA5* gene, which is responsible for fosfomycin resistance. Fosfomycin was known to be always effective in the treatment of MDR *Enterobacterales* (Falagas et al., 2010). However, such identification of the *fosA5* gene in one of *Enterobacterales* species; *K. pneumoniae* (K1), isolated mainly from raw milk, is considered a terrifying situation. Recently, some studies reported the fosfomycin resistance in isolates from beef samples (Sadek et al., 2020), human sputum (Soliman et al., 2020), and chicken feces (Soliman et al., 2021) in Egypt. This might be attributed to the habitual utilization of fosfomycin in combination with aminoglycosides widely in Egypt for the treatment of numerous diseases. Due to the misuse of different antimicrobial agents in Egypt, we found *K. pneumoniae* isolate was carrying not only the *mcr*-*10* and *fosA5* genes, but also multiple resistance genes to ß-lactamases, aminoglycosides, quinolones, tetracyclines, macrolides, and folate pathway inhibitors; unfortunately, these antimicrobials are used at both human and veterinary levels.

To correlate the genetic character of our K1 isolate with similar *K. pneumoniae* isolates worldwide, we performed a phylogenetic analysis to find out their epidemiological relationship.

Probably, we found that our isolate was clustered with 25 *K. pneumoniae* isolates from different sources including human, environment, and animal (Figure 3) supporting the direct/indirect transmission between human and animals and this in tune with the recent global concept of dissemination of mobilized colistin resistance genes from animals to humans (Luo et al., 2020). Followingly, this result emphasizing “the One Health” concept; human health is connected to the health of animals and the environment (Luo et al., 2020). Remarkably, this result affirms that *mcr* genes debilitate human wellbeing and contaminate the environment due to the plausibility of their worldwide spread brought from the raising, generation, worldwide exchange, and food utilization of animals (Anyanwu et al., 2020).

Similarly, when comparing the study isolate (K1) with different *mcr-10* harboring *K. pneumoniae*, our isolate presented in a clade consisting of isolates from numerous sources such as human and environment. Strikingly, this is the first identification of colistin *mcr*-*10* resistance gene in *K. pneumoniae* in the Middle East. Entirely, all formerly identified *mcr*-*10* carrying *K. pneumoniae* were detected in China, Europe, and Canada from clinical or environmental sources only. Given this close genetic distance between K1 isolate and the other *mcr*-*10* isolates (Figure 4), we investigated the sequence type of each isolate and they showed different sequence types. This agrees with what was reported by Luo et al. (2020), who documented that the sequence type diversity of the *mcr*-positive *E. coli* isolates showed a scattered and non-clonal prevalence.

## Conclusion

The emergence of *mcr*-*10* and *fosA5* harboring *K. pneumoniae* from raw milk is alarming and poses a public health threat. The existence of multiple resistance genes in MDR and XDR Gram-negative bacteria from dairy cattle provides evidence for its spread from the veterinary sector to humans and heralds the penetration of the last-resort antibiotics. Hence, prudent use of antibiotics in both humans and animals and antimicrobial surveillance plans are urgently required to fight XDR and MDR bacteria.

## Data Availability Statement

The data that support the findings of this study are available in the SRA database under the bioproject PRJNA713498.

## Ethics Statement

Ethical review and approval was not required for the animal study because this study did not include any experimental animals. Written informed consent was obtained from the owners for the participation of their animals in this study.

## Author Contributions

YHT and NKA contributed equally in the conception and design of the study and participated with RMAG and HME in the application of classical microbiological and molecular techniques. ME participated with HR in whole-genome sequence analysis. HR performed the bioinformatics. RMAG, HME, SE, EAS, ASAA, SSA, MMB, YAE-S, and ME conceived the study and participated in the design. RMAG, HME, SE, EAS, ASAA, SSA, MMB, YAE-S, and ME participated in interpretation of data. YHT, NKA, RMAG, HME, SE, and HR wrote the initial draft of the manuscript. All authors revised the manuscript critically for important intellectual content and gave the final approval of the version to be published.

## Conflict of Interest

The authors declare that the research was conducted in the absence of any commercial or financial relationships that could be construed as a potential conflict of interest.

## Publisher’s Note

All claims expressed in this article are solely those of the authors and do not necessarily represent those of their affiliated organizations, or those of the publisher, the editors and the reviewers. Any product that may be evaluated in this article, or claim that may be made by its manufacturer, is not guaranteed or endorsed by the publisher.

## Footnotes

1
https://www.bioinformatics.babraham.ac.uk/projects/fastqc/

2
http://www.usadellab.org/cms/index.php?page=trimmomatic

3
https://cab.spbu.ru/software/spades/

4
http://www.genomicepidemiology.org/services/

5
http://bacdb.cn/BacWGSTdb/

6
https://blast.ncbi.nlm.nih.gov/Blast.cgi

7
https://www-is.biotoul.fr/blast.php

8
http://mjsull.github.io/Easyfig/

9
http://sourceforge.net/projects/brig

10
https://github.com/tseemann/snippy
