# Supplementary material for: Whole-Genome Sequencing of Gram-Negative Bacteria Isolated From Bovine Mastitis and Raw Milk: The First Emergence of Colistin mcr-10 and Fosfomycin fosA5 Resistance Genes in Klebsiella pneumoniae in Middle East
Source: Front Microbiol. 2021 Dec 8;12:770813. doi: 10.3389/fmicb.2021.770813 (PMC8692987; doi:10.3389/fmicb.2021.770813)
Supplement: Supplementary file 1 [file Table_1.doc]

**Table S1:Oligonucleotide primer sequences used in this study**

| **Primer use and target gene** | **Nucleotide sequence (5′→3′)** | **Amplicon size (bp)** | **Annealing temperature (℃)** | **Reference** |
| --- | --- | --- | --- | --- |
|  |  |  |  |  |
| *E. coli uidA* | F: TATGGAATTTCGCCGATTTT  R: TGTTTGCCTCCCTGCTGCGG | 166 | 55 | [Heijnen](https://pubmed.ncbi.nlm.nih.gov/?term=Heijnen+L&cauthor_id=17176819)  and  [Medema](https://pubmed.ncbi.nlm.nih.gov/?term=Medema+G&cauthor_id=17176819)(2006) |
| *K. pneumoniae 16S-23S ITS* | F: ATTTGAAGAGGTTGCAAACGAT  R: TTCACTCTGAAGTTTTCTTGTGTTC | 130 | 55 | Liu et al. (2008) |
| *Citrobacter* species*16S rRNA* | F: GCTCAACCTGGGAACTGCATCCGA  R: AGTTCCGGCCTAACCGCTGGCAA | 529 | 58 | Anbazhagan et al. (2010) |
| *Enterobacter cloacae atpd* | F:CGAGAGCCTGGTGCTGC  R: GATTGGCTGACCCAAT | 180 | 58 | Anbazhagan et al. (2010) |
| *Proteus mirabilis ureR* | F: GGTGAGATTTGTATTAATGG  R: ATAATCTGGAAGATGACGAG | 255 | 58 | Zhang et al. (2013) |
| *Aeromonas hydrophila lip* | F: AACCTGGTTCCGCTCAAGCCGTTG  R: TTGCTCGCCTCGGCCCAGCAGCT | 1592 | 62 | Casco´n et al. (1996) |

F, forward; R, reverse; bp, base pair
